# Supplementary material for: Dietary Quality of Women of Reproductive Age in Low-Income Settings: A Cross-Sectional Study in Kyrgyzstan
Source: Nutrients. 2022 Jan 11;14(2):289. doi: 10.3390/nu14020289 (PMC8780699; doi:10.3390/nu14020289)
Supplement: Supplementary file 1 [file nutrients-14-00289-s001.zip › nutrients-1481155-supplementary.pdf]

Supplementary materials

We employed Kendall correlation tau to analyse correlation for categorical and continuous variables: DDS and socioeconomic and nutritional factors (age, education, family income, BMI level) (Table S1) as well as Wilcoxon test to compare continuous variables within groups (**DDS** with residence: rural/urban, region: North/South, remittances: yes/no, ethnicity: Kyrgyz/non-Kyrgyz, kitchen garden-cropland: yes/no, farm animals: yes/no, children: yes/no, farm harvest purpose: mostly sell/mostly self-consume, store distance: more than 2km/less than 2km) and found that some variables have no correlation and differences. Those variables which are statistically significant were included to the body of the article. DDS has no correlation and statistically insignificant with age, level of education, family income and BMI level (Table S1). DDS is not different among women receiving remittances and not receiving remittances, among Kyrgyz and non-Kyrgyz, among those who have children and who do not have one, among those who mostly sell their harvest and those who mostly consume themselves, and those who live close to the food market and those who live far from it (Table S2).

Table S1. Socioeconomic and demographic variables and DDS (Kendall tau correlation).

Variables	DDS tau	*p*	Adjusted *p*
Age:	0.04997285	0.222	0.8880000
Education:	-0.01829489	0.6535	0.9414667
Family income:	0.01482655	0.7061	0.9414667
BMI level:	-0.0008414101	0.9816	0.9816000

DDS – dietary diversity score

Table S2. Socioeconomic and demographic variables and DDS (Mann Whitney U test).

Variables	DDS<br>Cles, rbsc, pseudomedian, 95% CI low, 95% CI high, *p*-value	Adjusted *p*-value
Residence (Rural/urban)	0.3307487, -0.3385026, 0.999959, 2.228878e-05, 1.000043e+00, 1.368e-07*	0.0000012312**
Region (South/North)	0.6132317, 0.2264635, -3.735724e-05, -9.999299e-01, -1.496737e-05, 0.01431*	0.0321975000**
Remittances	0.491244, -0.01751193, 5.27875e-05, -4.678887e-05, 4.663804e-05, 0.3411	0.4385571429
Ethnicity	0.4716438, -0.05671233, -2.005951e-05, -5.213814e-05, 3.624930e-05, 0.4389	0.4937625000
Kitchen garden	0.5986375, 0.1972751, -0.999971, -9.999430e-01, -4.461354e-05, 0.0002611*	0.0011749500**
Farm animals	0.598561, 0.1971219,	0.0093060000**

	-4.22736e-05,<br>-9.999390e-01,<br>-4.672716e-05, 0.003102*	
Children	0.5688372, 0.1376743, -5.093497e-05, -5.526166e-05, 7.103729e-05, 0.1968	0.2952000000
Harvest animal purpose	0.5610867, 0.1221734, -9.703766e-05, -9.999339e-01, 3.130242e-05, 0.4965	0.4965000000
Store distance	0.5528687, 0.1057375, -2.868532e-06, -0.9999854204, 0.0000538829, 0.1809	0.2952000000

DDS – dietary diversity score; Cles – common language effect size; rbsc – rank-biserial correlation

We employed Kendall tau correlation for continuous and categorical variables (nutrient adequacy ratios for different macro- and micronutrients and variables: income, education, and BMI (Table S3). The Results section of the article contains details of findings regarding this table with a figure.

Table S3. Relationships between NAR for macro- and micronutrients and some socioeconomic indicators.

**Variables**	**Income**			**Education**			**BMI**		
	**Kendall's correlation (tau)**	***p*-value**	**adjusted *p*-value**	**Kendall's correlation (tau)**	***p*-value**	**adjusted *p*-value**	**Kendall's correlation (tau)**	***p*-value**	**adjusted *p*-value**
NAR energy	-0.053	0.146	0.310	-0.119	0.001*	0.006**	0.03	0.376	0.436
NAR protein	-0.045	0.216	0.4003	-0.048	0.204	0.247	0.019	0.556	0.590
NAR fat	0.017	0.644	0.684	-0.0305	0.416	0.442	-0.004	0.894	0.892
NAR carbohydrates	-0.075	0.037*	0.152	-0.138	0.0002*	0.001**	0.050	0.125	0.255
NAR fibre	-0.133	0.0002*	0.003**	-0.170	0.059*	0.0001**	0.094	0.003*	0.080
NAR Vit. A	-0.043	0.235	0.4003	-0.065	0.084	0.129	0.049	0.128	0.255
NAR Vit E.	0.006	0.871	0.871	-0.073	0.050*	0.085	0.057	0.082	0.255
NAR Vit B1	-0.076	0.044*	0.152	-0.104	0.008*	0.016**	0.042	0.222	0.314
NAR Vit B2	-0.037	0.319	0.494	-0.053	0.173	0.244	0.024	0.478	0.541
NAR Vit B6	-0.017	0.643	0.684	-0.049	0.204	0.247	0.045	0.182	0.309

NAR fol. acid	-0.113	0.001*	0.015**	-0.153	0.047*	>0.001**	0.058	0.078	0.255
NAR Vit. C	0.018	0.615	0.684	-0.018	0.622	0.622	0.033	0.314	0.411
NAR calcium	-0.031	0.392	0.512	-0.075	0.046*	0.085	0.049	0.135	0.255
NAR magnesium	-0.075*	0.038*	0.152	-0.102	0.007*	0.016**	0.055	0.094	0.255
NAR iron	-0.059	0.107	0.272	-0.113	0.002*	0.008**	0.053	0.108	0.255
NAR zink	-0.034	0.349	0.494	-0.041	0.272	0.308	0.040	0.215	0.314
MAR 16	-0.058	0.112	0.272	-0.111	0.003*	0.008**	0.063	0.054	0.255

NAR – nutrient adequacy ratio; Mar 16 – mean adequacy ratio; *p-value < 0.05; **adjusted p-value < 0.05.

We used Mann Whitney U test to continuous and binary variables: NARs for macro- and micronutrients and residence: rural/urban, kitchen garden-cropland: yes/no, harvest animal purpose: mostly sell/mostly self-consume, store distance: less than 2km/more than 2km, remittances: yes/no, farm animal: yes/no (Table S4). Results are discussed in the Results section of the paper.

Table S4. Factors determining diet adequacy.

**Variables**	**Residence**	**Kitchen garden/cropland**	**Harvest animal purpose**	**Store distance**	**Remittances**	**Farm animal**
**Cles, rbsc, pseudomedian, 95% CI low, 95% CI high, *p*-value, *p* value adjusted**						
NAR energy	0.4178735,<br>-0.1642529,<br>0.08002865,<br>0.02992904,<br>0.13995894,<br>0.002504*<br>1.064200e-02**	0.4297417,<br>-0.1405166,<br>0.07003125,<br>0.02004382,<br>0.12996596,<br>0.006965*<br>0.029601250**	0.4608166,<br>-0.07836685,<br>0.03000906,<br>-0.04003885,<br>0.10998086,<br>0.367<br>0.7798750	0.564182,<br>0.128364,<br>-0.06004081,<br>-0.14992098,<br>0.02997664,<br>0.1775<br>0.2953750	0.5555778,<br>0.1111555,<br>-0.06006198,<br>-0.1299710768,<br>0.0000719134,<br>0.06681<br>0.14197125	0.5822907,<br>0.1645814,<br>-0.08003687,<br>-0.13998215,<br>-0.03000136,<br>0.002615*<br>0.0148183333**
NAR protein	0.5379089,<br>0.07581789,<br>-0.03000421,<br>-0.10996923,<br>0.04996492,<br>0.4502<br>6.951692e-01	0.4826845,<br>-0.03463097,<br>0.02003718,<br>-0.04995318,<br>0.09998156, 0.5131<br>0.670976923	0.4397473,<br>-0.1205054,<br>0.0599629,<br>-0.04001165,<br>0.15998980,<br>0.2667<br>0.7556500	0.5883583,<br>0.1767165,<br>-0.08000158,<br>-0.19000683,<br>0.03005915,<br>0.1638<br>0.2953750	0.5424521,<br>0.08490415,<br>-0.07994904,<br>-0.16001741,<br>0.01008866,<br>0.08552<br>0.16153778	0.5189268,<br>0.03785353,<br>-0.03001758,<br>-0.10002434<br>0.04001866,<br>0.4013<br>0.5685083333
NAR fat	0.482819,<br>-0.03436201,<br>0.02995502,<br>-0.05999925,<br>0.10000954,<br>0.5262	0.4972932,<br>-0.005413541,<br>0.009970382,<br>-0.07007149,<br>0.08998467, 0.8438<br>0.896537500	0.4601262,<br>-0.0797475,<br>0.05002971,<br>-0.06004967,<br>0.15996399,<br>0.3622	0.5729579,<br>0.1459158,<br>-0.1099964,<br>-0.230070458,<br>0.009936597,<br>0.06188	0.5157661,<br>0.03153213,<br>-0.03997604,<br>-0.13002569,<br>0.05997462,<br>0.4232	0.4941409,<br>-0.01171811,<br>0.01998628,<br>-0.06998256<br>0.09004553,<br>0.7167

	6.951692e-01		0.7798750	0.2953750	0.51388571	0.8905571429
NAR carbohy-drate	0.3670179,<br>-0.2659642,<br>0.1299165,<br>0.07994871,<br>0.18001836,<br>3.743e-06*<br>3.181550e-05**	0.3921873,<br>-0.2156254,<br>0.09999568,<br>0.04997043,<br>0.15995385,<br>0.0003443*<br>0.002926550**	0.4850633,<br>-0.02987346,<br>0.009969848,<br>-0.07005244,<br>0.08002656,<br>0.8737<br>0.9283062	0.5230399,<br>0.04607982,<br>-0.02999155,<br>-0.12993219,<br>0.06993472,<br>0.5597<br>0.6343267	0.5666272,<br>0.1332544,<br>-0.06994448,<br>-1.399870e-01,<br>-7.063806e-05,<br>0.04013*<br>0.09903714	0.6322409,<br>0.2644819,<br>-0.1299886,<br>-0.18998193<br>-0.07000424,<br>7.401e-06<br>0.0000629085**
NAR fibre	0.3783776,<br>-0.2432448,<br>0.1500047,<br>0.08993393,<br>0.21994411,<br>8.539e-06<br>4.838767e-05**	0.3936846,<br>-0.2126307,<br>0.1399684,<br>0.0700439,<br>0.2000470, 5.859e-05<br>0.000996030**	0.4728676,<br>-0.05426489,<br>0.01003457,<br>-0.08003811,<br>0.10995617,<br>0.7823<br>0.9283062	0.5191042,<br>0.03820835,<br>-7.792522e-06,<br>-0.1199429,<br>0.1100440,<br>0.9674<br>0.9704000	0.5748546,<br>0.1497092,<br>-0.09006704,<br>-0.17001565<br>-0.01006266,<br>0.02169*<br>0.08785600	0.6305862,<br>0.2611723,<br>-0.170049,<br>-0.2400624<br>-0.1099793,<br>8.956e-07<br>0.0000152252**
NAR Vit. A	0.4701644,<br>-0.05967114,<br>0.0199919,<br>-0.07993667,<br>0.12005190,<br>0.6934<br>8.419857e-01	0.4718786,<br>-0.05624288,<br>0.0399634,<br>-0.06000363,<br>0.13997703,<br>0.4632<br>0.656200000	0.4813813,<br>-0.0372374,<br>0.1200573,<br>-0.02005334,<br>0.28000108,<br>0.1056<br>0.7556500	0.5131686,<br>0.02633715,<br>4.334691e-05,<br>-0.1500727,<br>0.1699653,<br>0.9704<br>0.9704000	0.4906194,<br>-0.01876112,<br>-0.029949,<br>-0.15008539,<br>0.09999701,<br>0.6653<br>0.73631250	0.5268961,<br>0.05379226,<br>3.208944e-06,<br>-0.1099184<br>0.1000354,<br>0.9698<br>0.9698000000
NAR Vit E.	0.5388153,<br>0.07763066,<br>-0.05993001,<br>-0.14996093,<br>0.02999586,<br>0.1875<br>3.541667e-01**	0.5291968,<br>0.0583935,<br>-0.01995276,<br>-0.10992268,<br>0.06000014, 0.6326<br>0.768157143	0.4906868,<br>-0.01862632,<br>0.03007947,<br>-0.07002139,<br>0.13997085,<br>0.5584<br>0.9283062	0.5746354,<br>0.1492709,<br>-0.07001299,<br>-0.20997791<br>0.04997141,<br>0.2085<br>0.2953750	0.4888186,<br>-0.02236272,<br>-0.00996704,<br>-0.10000257<br>0.09005944,<br>0.8957<br>0.89570000	0.4593301,<br>-0.08133985,<br>0.05997089,<br>-0.02001871<br>0.14001980,<br>0.1639<br>0.3095888889
NAR Vit B1	0.4722096,<br>-0.05558084,<br>3.668929e-05,<br>-6.512014e-05,<br>6.992468e-02,<br>0.09382<br>2.278486e-01**	0.4638614,<br>-0.07227722,<br>0.05995351,<br>2.982409e-05,<br>6.998800e-02,<br>0.01321*<br>0.044914000**	0.4567277,<br>-0.08654454,<br>5.650447e-05,<br>-0.0000328337,<br>0.0600211387,<br>0.6732<br>0.9283062	0.5899683,<br>0.1799366,<br>-8.015769e-05,<br>-0.0700266073,<br>0.0000330382,<br>0.1754<br>0.2953750	0.5654107,<br>0.1308213,<br>-0.06004653,<br>-7.001561e-02,<br>-5.006409e-05,<br>0.007304*<br>0.08785600	0.5259195,<br>0.05183891,<br>-5.620605e-06,<br>-6.998033e-02<br>5.826962e-06,<br>0.1205<br>0.2560625000
NAR Vit B2	0.4926454,<br>-0.0147092,<br>-1.010764e-05,<br>-0.04998924,<br>0.04997835,<br>0.8735<br>9.280938e-01	0.469201,<br>-0.06159806,<br>2.81072e-05,<br>-2.021424e-05,<br>5.002721e-02,<br>0.3797<br>0.586809091	0.5220457,<br>0.04409141,<br>3.779491e-05,<br>-0.04996107,<br>0.05000760,<br>0.7773<br>0.9283062	0.6090998,<br>0.2181996,<br>-0.05993355,<br>-1.099534e-01,<br>-4.770645e-05,<br>0.02075*<br>0.2299250	0.5385477,<br>0.07709533,<br>-0.04996032,<br>-5.998548e-02<br>3.271315e-05,<br>0.09897<br>0.16824900	0.513593,<br>0.0271859,<br>-1.444069e-05,<br>-0.04996620<br>0.04997417,<br>0.9202<br>0.9698000000
NAR Vit B6	0.5067021,<br>0.01340424,<br>3.238713e-05,<br>-0.04997802,<br>0.049964600,<br>0.9283<br>9.283000e-01	0.481279,<br>-0.03744196,<br>3.977365e-05,<br>-6.096123e-06,<br>5.002696e-02,<br>0.3011<br>0.511870000	0.4723258,<br>-0.05534843,<br>9.286e-06, -<br>0.04998214,<br>0.05006314,<br>0.8527<br>0.9283062	0.6211364,<br>0.2422728,<br>-0.05004067,<br>-1.000311e-01,<br>-7.145601e-05,<br>0.02705*<br>0.2299250	0.518163,<br>0.03632607,<br>-3.577752e-05,<br>-5.001774e-02<br>1.883423e-05,<br>0.3214<br>0.45531667	0.5176403,<br>0.0352805,<br>-4.035236e-05,<br>-5.002502e-02<br>6.566271e-05,<br>0.3823<br>0.5685083333
NAR fol. acid	0.4291592,	0.456799,	0.5740547,<br>0.1481093,	0.5773688,<br>0.1547375,	0.5688198,<br>0.1376396,	0.5383022,<br>0.07660434,

	-0.1416815,<br>0.3949441,<br>0.3749520,<br>0.4150419,<br>2.2e-16*<br>3.740000e-15**	-0.08640206,<br>0.04993223,<br>0.01996194,<br>0.07997687,<br>0.001546*<br>0.008760667**	-0.03995732,<br>-7.996184e-02,<br>1.224565e-05,<br>0.07847<br>0.7556500	-0.03001781,<br>-0.07993603,<br>0.01998416,<br>0.2038<br>0.2953750	-0.04996832,<br>-0.080008068,<br>-0.009999027,<br>0.01591*<br>0.08785600	-0.04000553,<br>-0.07003246<br>-0.01002450,<br>0.006049*<br>0.0257082500**
NAR Vit. C	0.5172243,<br>0.03444858,<br>-0.009958969,<br>-0.05997019,<br>0.04999773,<br>0.8386<br>9.280938e-01	0.5136736,<br>0.02734716,<br>4.266827e-05,<br>-0.05004070,<br>0.05993263,<br>0.9353<br>0.935300000	0.4695681,<br>-0.06086374,<br>-2.387871e-05,<br>-0.07991099<br>0.07003053,<br>0.9667<br>0.9667000	0.568728,<br>0.137456,<br>-0.05005258,<br>-0.14006239,<br>0.02998933,<br>0.1906<br>0.2953750	0.5350799,<br>0.07015974,<br>-0.01004382,<br>-0.07991553,<br>0.05000550,<br>0.693<br>0.73631250	0.4919568,<br>-0.01608646,<br>1.070791e-06,<br>-0.05996945<br>0.05000863,<br>0.9624<br>0.9698000000
NAR calcium	0.4309787,<br>-0.1380425,<br>0.02993532,<br>1.094691e-05,<br>4.997193e-02,<br>0.02626*<br>8.928400e-02	0.4585224,<br>-0.08295526,<br>0.01997549, -<br>1.734711e-05,<br>4.004132e-02,<br>0.1134<br>0.214200000	0.5592598,<br>0.1185196,<br>-0.0100365,<br>-0.04002861,<br>0.01999683,<br>0.4376<br>0.8265778	0.5663869,<br>0.1327737,<br>-0.02002771,<br>-0.05995206,<br>0.01004624,<br>0.2311<br>0.3022077	0.5154893,<br>0.03097866,<br>-0.01003646,<br>-0.03996898,<br>0.01991966,<br>0.423<br>0.51388571	0.5261117,<br>0.05222343,<br>-0.0100152,<br>-0.03999229<br>0.01004212,<br>0.2919<br>0.4962300000
NAR magne-<br>sium	0.4527854,<br>-0.09442921,<br>0.03999853,<br>5.315912e-05,<br>7.005021e-02,<br>0.04368*<br>1.237600e-01**	0.4560631,<br>-0.08787387,<br>0.03994473,<br>1.012004e-05,<br>7.004725e-02,<br>0.03067*<br>0.086898333	0.5302609,<br>0.06052178,<br>-7.887231e-06,<br>-0.04001535,<br>0.03999851,<br>0.8719<br>0.9283062	0.6083082,<br>0.2166163,<br>-0.04005839,<br>-9.998112e-02,<br>3.650134e-05,<br>0.07915<br>0.2953750	0.5515902,<br>0.1031803,<br>-0.03001889,<br>-0.07002896<br>0.01006845,<br>0.1383<br>0.21373636	0.5429556,<br>0.08591115,<br>-0.03003839,<br>-6.993784e-02<br>6.299116e-05,<br>0.05326<br>0.1810840000
NAR iron	0.4903699,<br>-0.0192602,<br>0.01002895,<br>-0.02992368,<br>0.05999170,<br>0.5316<br>6.951692e-01	0.4576825,<br>-0.08463494,<br>0.04003451,<br>-5.831504e-05,<br>8.001718e-02,<br>0.06106<br>0.129752500	0.4554134,<br>-0.08917315,<br>0.03004174,<br>-0.02006113,<br>0.08999049,<br>0.2458<br>0.7556500	0.5931251,<br>0.1862501,<br>-0.04997049,<br>-0.10998998,<br>0.01002239,<br>0.13,<br>0.2953750	0.5633498,<br>0.1266995,<br>-0.06000465,<br>-0.11002263,<br>-0.01002358,<br>0.01575*<br>0.08785600	0.5403565,<br>0.08071305,<br>-0.0300369,<br>-0.079966159<br>0.009996825,<br>0.1079<br>0.2560625000
NAR zink	0.5590078,<br>0.1180155,<br>-0.04999226,<br>-0.1200016,<br>0.0200065,<br>0.168<br>3.541667e-01**	0.4952984,<br>-0.009403167,<br>0.01004748,<br>-0.05995692,<br>0.07999270, 0.7207<br>0.816793333	0.4357427,<br>-0.1285146,<br>0.04995845,<br>-0.03001893,<br>0.13999303,<br>0.2477<br>0.7556500	0.5777312,<br>0.1554624,<br>-0.05002858,<br>-0.15996314,<br>0.04998168,<br>0.3187<br>0.3869929	0.5514442,<br>0.1028885,<br>-0.08999031,<br>-0.16003969,<br>-0.01007552,<br>0.02584*<br>0.08785600	0.5009113,<br>0.001822621,<br>-0.01003949,<br>-0.07994275<br>0.05998439,<br>0.7334<br>0.8905571429
MAR 16	0.4699761,<br>-0.06004777,<br>0.02996721,<br>-0.01996689,<br>0.06996626,<br>0.2542<br>4.321400e-01**	0.459366,<br>-0.08126807,<br>0.04004667,<br>7.53816e-05,<br>8.99641e-02,<br>0.04499*<br>0.109261429	0.4653376,<br>-0.06932484,<br>0.03995661,<br>-0.01993707,<br>0.09008634,<br>0.2086<br>0.7556500	0.5918606,<br>0.1837213,<br>-0.05003699,<br>-0.120067213,<br>0.009959691,<br>0.08688<br>0.2953750	0.5481874,<br>0.09637477,<br>-0.05002088,<br>-1.099926e-01,<br>-1.045352e-05,<br>0.04078*<br>0.09903714	0.5412512,<br>0.08250239,<br>-0.04004113,<br>-8.006802e-02<br>7.885911e-06,<br>0.07299<br>0.2068050000

NAR – nutrient adequacy ratio; Mar 16 – mean adequacy ratio; Cles – common language effect size; rbsc – rank-biserial correlation; *p-value < 0.05; **adjusted p-value < 0.05.
